# Changes in soil bacterial community structure as a result of incorporation of *Brassica* plants compared with continuous planting eggplant and chemical disinfection in greenhouses

**DOI:** 10.1371/journal.pone.0173923

**Published:** 2017-03-27

**Authors:** Tianzhu Li, Tongtong Liu, Chengyu Zheng, Chunsheng Kang, Zichao Yang, Xiaotong Yao, Fengbin Song, Runzhi Zhang, Xuerong Wang, Ning Xu, Chunyi Zhang, Wei Li, Shumin Li

**Affiliations:** 1 Resource and Environmental College, Northeast Agricultural University, Harbin, China; 2 Research Development and Evaluation Center of Laboratory, Harbin, China; Universita degli Studi di Pisa, ITALY

## Abstract

Greenhouse eggplant monocropping in China has contributed to the aggravation of soil-borne diseases, reductions in crop quality and yield, and the degradation of physical and chemical soil properties. Crop rotation is one effective way of alleviating the problems of continuous cropping worldwide; however, few studies have reported changes in soil bacterial community structures and physical and chemical soil properties after *Brassica* vegetables had been rotated with eggplant in greenhouses. In this experiment, mustard-eggplant (BFN) and oilseed rape-eggplant (BFC) rotations were studied to identify changes in the physicochemical properties and bacterial community structure in soil that was previously subject to monocropping. Samples were taken after two types of *Brassica* plants incorporated into soil for 15 days to compare with continually planted eggplant (control, CN) and chemical disinfection of soil (CF) in greenhouses. MiSeq pyrosequencing was used to analyze soil bacterial diversity and structure in the four different treatments. A total of 55,129 reads were identified, and rarefaction analysis showed that the soil treatments were equally sampled. The bacterial richness of the BFC treatment and the diversity of the BFN treatment were significantly higher than those of the other treatments. Further comparison showed that the bacterial community structures of BFC and BFN treatments were also different from CN and CF treatments. The relative abundance of several dominant bacterial genera in the BFC and BFN treatments (such as *Flavobacteria*, *Stenotrophomonas*, *Massilia* and *Cellvibrio*, which played different roles in improving soil fertility and advancing plant growth) was distinctly higher than the CN or CF treatments. Additionally, the total organic matter and Olsen-P content of the BFC and BFN treatments were significantly greater than the CN treatment. We conclude that *Brassica* vegetables-eggplant crop rotations could provide a more effective means of solving the problems of greenhouse eggplant monocultures.

## Introduction

A common phenomenon in eggplant (*Solanum melongena* L.) cropping systems is “continuous monoculture”, which means growing the same crop year after year in the same field. This practice exists in China due to the opposing forces of high market demand and limited cultivated land [1]. However, a series of problems result from the eggplant monoculture in China, such as the aggravation of soil-borne disease, reductions in crop quality and yield [2], and degraded soil physical and chemical properties [3]. In the Mediterranean region, the continuous cropping of eggplant has had negative effects on soil and plant growth due to an uneven supply of nutrients and increasingly widespread populations of nematodes and soil-borne pathogens [4]. Vanlauwe et al. (2001) found that there was an increase in soil acidification when nitrogen fertilizers were used excessively, affecting the microbial diversity of the soil. Long term monoculture with a single plant also harmed the rhizosphere of crop microorganisms [5] and eliminated soil biological diversity. Soil is a complex and dynamic environment, and its biological activity is mostly governed by microorganisms [6]. Continuous cropping alters soil composition and microbial community structures [7] which are important indicators of soil fertility [8].

Crop rotation is an effective way to alleviate problems with continuous cropping worldwide. If rotated crops are selected properly, the effectiveness of rotation can be maximized. Tian et al. (2009) found that in both tomato-onion and chrysanthemum-tomato rotations, tomato yield, organic C, total N and total microbial population significantly improved compared with continuous planting and were positively correlated [9]. Winter cover crops (oilseed rape) and their residue were beneficial to eggplant growth; eggplant yield increased by 46.19% in 2011 since nitrogen utilization efficiency was enhanced [10, 11]. Rice-rapeseed and cotton-rapeseed crop rotations further improved N-use efficiency and increased the yields of seeds from previous crops [12]. The tissues of the rotated vegetables are beneficial to the elemental composition of the soil, subsequently improving plant growth. Therefore, crop soil quality could be improved by selecting crops based on their chemical composition to influence soil carbon content [13].

Furthermore, incidences of soil-borne diseases can be reduced by influencing microbial structures and its activities [14]. Therefore, it is important to explore how strongly crop rotations alter the diversity and composition of bacterial and fungal communities [15]. Several beneficial bacterial strains isolated from the rhizosphere of *Brassica* species [16] are associated with plant growth and promoting rhizobacteria (PGPR), such as *Agrobacterium*, *Azospirillum*, *Bacillus*, *Enterobacter*, *Pseudomonas* and *Rhizobium* [17]. These microorganisms can promote plant growth by regulating nutritional and hormonal balances, producing plant growth regulators [18], solubilizing nutrients and inducing resistance against plant pathogens [19]. Some studies have shown that the incorporation of *Brassica* crops into soil inhibited the growth of a variety of soil-borne pathogens of potato, including *Rhizoctonia solani*, *Phytophthora erythroseptica*, *Pythium ultimum*, *Sclerotinia sclerotiorum* and *Fusarium sambucinum*, and reduced subsequent potato seedling disease by 40–83% [20]. Microbial diversity is related to soil function and ecosystem sustainability; therefore, it could influence the incidence of soil disease [21]. For example, *Streptomyces* strains can suppress the growth of *Rhizoctonia solani* in soil. When *Brassica* tissues are incorporated into the soil and decomposed, the populations of antagonistic bacteria (*Streptomyces* strains) increased, which could decrease the quantity of *R*. *solani* [22, 23].

Mustard and oilseed rape growing in autumn with short growth periods and large biomasses are common vegetables in China. In contrast, eggplant grows in the summer months. Therefore, we selected these *Brassica* plants for the eggplant rotation, so that their function as soil disinfectants could be performed most effectively in the greenhouse environment. Because *Brassica* vegetables contain high concentrations of glucosinolates, which breakdown products such as isothiocyanate [24] have the function of killing soil bacteria due to their toxicity [25].

Above all, the disinfectants could affect the composition of soil microbial communities and the rates of soil processes, such as C and N mineralization. Thus, the pH value and available N, P and K contents in soil would be changed [26]. High throughput sequencing is known to be a powerful tool for studying bacterial communities that has been widely used in numerous studies [4, 27]. Zhang et al. (2015) effectively used MiSeq high throughput sequencing to provide insight into the diversity of bacterial communities with specific primers at a fine scale structure [28].

Heilong Jiang Province is located in the alpine zone, where mustard, oilseed rape and eggplant are common crops. However, *Brassica* vegetable-eggplant rotations have rarely been studied. Therefore, this study’s objective was to evaluate the physicochemical and biological effects of these two *Brassica* vegetables within a greenhouse eggplant rotation compared with eggplant monocropping. Soil physical and chemical properties were examined, along with bacterial diversity and community structure.

## Materials and methods

### Experimental design

This experiment was conducted in a plastic greenhouse at the Horticultural Institute in Heilong Jiang Province, China. Heilong Jiang Province is located at north latitude 45° 45′, where the greenhouse growing season of eggplant is usually from May to August and where eggplant is planted once a year. The greenhouse soil that we used to do the experiment was black soil that had been continuously planted with eggplant for 13 years. Since we studied the challenges of eggplant monocropping, no short crop was planted after eggplant harvesting. The disease incident rate of eggplants in this greenhouse had reached 70% or more in each growing season during previous several years, with a predominant occurrence of *Verticillium dahlia*.

In this experiment, we designed four treatments with three replications, twelve plots were in a completely randomized design. The treatments included continuous eggplant (denoted by CN), eggplant-mustard rotation (*Brassica napiformis* var. ErDao mei, denoted by BFN), eggplant-oilseed rape rotation (*Brassica campestris* L. WoGuan-2, denoted by BFC) and soil disinfected by chemical pesticide (denoted by CF). Each plot was 5 m (width) ×10 m (length) with a 1 m interval between two plots to avoid cross-contact among the different treatments. Mustard and oilseed rape seeds were planted on August 18, 2015. Forty five days after sowing, mustard (BFN) and oilseed rape (BFC) plants were incorporated into the soil and mixed completely to 20 cm depth by a rotary machine (garden equipment) on October 16, 2015. At the same time, the soil was fumigated in CF treatment plot using150 g 50% carbendazim WP (Jiangsu Lanfeng Biological Chemical Co., Ltd.). Then, each plot was irrigated until 75% of the soil was saturated, and was covered with plastic film. After fumigating for 15 days, soil samples were collected from ten points in each plot plow layer (0–20 cm) according to a staggered grid method using a soil auger. Soil samples from their respective plots were mixed separately and immediately stored in an ice box. After these soil samples were taken back to the laboratory half of each sample was air-dried for measuring soil physical and chemical properties, and the remainder was stored in -80°C refrigerator for soil DNA extraction.

### Determination of soil physicochemical characteristics

The physical and chemical properties of soil were determined following Shidan Bao’s method (Agrochemical soil analysis book, 2005). Part of the air-dried soil was using a 20-mesh sieve, and the rest of the soil was dried and set aside using a 100-mesh sieve. Soil through the 20-mesh sieve was used to measure soil pH and available P and K. The soil pH value was determined in a soil-water suspension (1:1 ratio) with glass electrodes. Available P (Olsen-P) was extracted with a 0.5 mol•L^-1^ NaHCO_3_ solution and then measured using molybdenum-antimony-D-isoascorbic-acid-colorimetry (MADAC) at 880 nm. Available K was measured by flame photometry after 1 mol•L^-1^ NH_4_OAc neutral extraction. Soil through the 100-mesh sieve was used to measure organic matter and total N. The level of organic matter in the soil was determined by volumetric analysis with a heated K_2_Cr_2_O_7_ solution. Total soil nitrogen was measured using the Kjeldahl method with heated sulfuric acid concentrate. The method used to measure the total phosphorus in the soil was the same method used to measure available P. Fresh soil was used to determine both NO_3_^-^-N and NH_4_^+^-N, which were extracted using 2.0 mol•L^-1^ KCl in a soil-water suspension (a 1:5 ratio) and measured with a Bran-Luebbe Auto Analyzer 3 (Germany). The treatments and the soil physicochemical characteristics of each sample are summarized in Table 1.

**Table 1:** Soil physicochemical characteristics of different treatments.

Treatments	pH	Organic matter	Organic C	Total N	Total P	Available P	Available K	Ammonium N	Nitrate N
		(g•kg^-1^)	(g•kg^-1^)	(g•kg^-1^)	(g•kg^-1^)	(mg•kg^-1^)	(mg•kg^-1^)	(mg•kg^-1^)	(mg•kg^-1^)
CN	7.07 b	20.42 b	11.84 b	1.41 ab	2.33 b	128.63 c	187.69 a	14.08 a	32.77 a
BFN	7.47 a	53.30 a	30.92 a	1.31 b	2.63 a	138.53 b	186.83 a	13.68 a	28.91 b
BFC	7.39 a	43.67 a	25.33 a	1.31 b	2.17 c	182.67 a	179.19 b	13.92 a	14.03 c
CF	7.07 b	27.62 b	16.02 b	1.44 a	2.30 b	131.57 c	187.18 a	14.02 a	32.83 a

Note: Mean values (n = 3) for each treatment that are followed by the same letter are not significantly different (P ≤0.05) between treatments in the same column.

### DNA extraction

Before DNA extraction, visible plant roots were carefully removed from each sample using sterile tweezers. Soil microbial DNA was isolated using a Fast DNA^®^ Spin Kit for Soil (MP Bio medicals, U.S.A) per the manufacturer’s instructions. The DNA extracts were stored at -20°C for PCR amplification. PCR amplification was performed in an ABI Gene Amp^®^ 9700 (USA). The PCR experiments used a Trans Gen AP221-02: Trans Start Fastpfu DNA Polymerase, 20 μl reaction system involving 4 μl 5×FastPfu Buffer, 2 μl 2.5 mM dNTPs, 0.8 μl Forward Primer (5 μM), 0.8 μl reverse primer (5 μM), 0.4 μl FastPfu Polymerase, 0.2 μl BSA, 10 ng Template DNA and a slight amount of ddH_2_O, resulting in a total volume of 20 μl. The V4-V5 region of the 16S ribosomal RNA bacterial gene was amplified by PCR using primers 338F (5′-ACTCCTACGGGAGGCAGCAG-3′) and 806R (3′-TACHVGGGTWTCTAAT-5′), where the barcode is an eight-base sequence unique to each sample. For Part I, we performed a 3 min denaturation at 95°C. For Part II, we completed 27 cycles of denaturing for 30 s at 95°C, annealed for 30 s at 55°C and extended for 45 s at 72°C. Part III, involved a final extension for 10 min at 72°C.

### Illumina MiSeq sequencing

The PCR products that were used consisted of 2% agarose gel electrophoresis, and purified using a Axy Prep DNA Gel Evulsion Kit (Axygen Company) Gel Extraction PCR product eluted using Tris-HCl and were quantified using QuantiFluor TM-ST (Promega, U.S.). Purified amplicons were sequenced on an Illumina MiSeq Sequencer according to the standard recommendations. The raw reads were deposited into the NCBI Sequence Read Archive database.

### Processing of sequenced data

Demultiplex Raw fastq files were quality-filtered by QIIME (version 1.17).

OTUs were clustered with a 97% similarity cutoff by UPARSE (version 7.1 http://drive5.com/uparse/) and chimeric sequences were identified and removed by UCHIME.

The taxonomy of the 16S rRNA gene sequence was analyzed by an RDP Classifier (http://rdp.cme.msu.edu/) against the SILVA (SSU115)16S rRNA database using a confidence threshold of 70% [29].

Rarefaction data and Simpson, Shannon-Weaver and Chao indices were created using Mothur (Version 1.36.1) [30].

### Statistical analysis

All statistical analyses were performed using SPSS statistical software (IBM SPSS Statistics 19). Differences at the P ≤ 0.05 level was considered to be statistically significant.

## Results

### Change of soil physicochemical characteristics

Fifteen days after mustard (BFN) and oilseed rape (BFC) plants incorporated into soil, the soil pH of the BFN and BFC treatments increased compared with the CN and CF treatments (Table 1). The soil organic matter of the BFN treatment was 2.65 times greater than the CN treatment. Soil available P content of BFC was 54.04 mg•kg^-1^ higher than that of the CN treatment. Compared with the control, the total P of the BFN treatment increased 0.30 g by adding mustard, but the phosphorus level dropped 0.16 g by adding oilseed rape in the BFC treatment. However, total nitrogen, nitrate and soil available K concentrations of the BFN and BFC treatments were significantly lower than that of CN and CF. In addition, there was no significant difference in soil ammonium between the different treatments.

### Bacterial diversity and community structures

The gene sequence information from the samples and the calculated bacterial richness and diversity indices are listed in Table 2. There were 55,129 reads of the 16S rRNA gene which were extracted from 12 samples of the four treatments that represented the wide diversity of the bacterial community. These reads included 4,120 OTUs belonging to 39 phyla, 92 classes, 201 orders, 386 families, 741 genera, and 1,485 species. The sequence information can be seen in Table 2, with a cutoff of 3% of the bacterial richness and diversity index among the four different treatments. Ace and Chao values represent the richness index. The BFC treatment had the largest richness value, while the index of the CF treatment was the smallest. These results indicate that the bacterial richness of the BFC treatment was the highest, the bacterial richness of the CF treatment was the lowest. A larger Shannon-Weaver index indicates higher diversity, while the Shannon-Weaver and Simpson indices are inversely proportional. The Shannon-Weaver index varied between 6.47 and 7.05. The largest value of the Shannon-Weaver index was found in the CN treatment, and the lowest one was in the BFN. However, the Simpson index varied between 0.0020 and 0.0076, with the largest value for the BFN and the lowest one for the CN. These results indicate that bacterial diversity in the rotational treatments was higher than in the CF and CN treatments. In all, the richness and diversity of bacteria increased with mustard-eggplant and oilseed rape-eggplant rotations.

**Table 2:** Bacterial diversity indices of different rotational treatments.

Treatments	Reads	0.97				
		OTU	Ace	Chao	Shannon	Simpson
BFC1	55129	3415	3773	3801	6.95	0.0029
BFC2		3500	3854	3860	7.00	0.0029
BFC3		3463	3753	3740	7.09	0.0021
BFC_Ave_^1^		10378	3793 a^2^	3800 a	7.01 a	0.0026 a 0.0039
BFN1		3350	3773	3744	6.78	
BFN2		3024	3629	3607	5.87	0.015
BFN3		3310	3738	3758	6.76	0.0036
BFN_Ave_		9684	3713b	3703 a	6.47 b	0.0076 a
CF1		3307	3684	3724	6.98	0.0024
CF2		3230	3682	3767	6.86	0.0033
CF3		3235	3581	3634	6.96	0.0027
CF_Ave_		9772	3649 b	3708 a	6.93 ab	0.0028 a
CN1		3333	3710	3751	7.04	0.0020
CN2		3363	3699	3720	7.03	0.0022
CN3		3442	3771	3814	7.09	0.0019
CN_Ave_		10138	3727 ab	3762 a	7.05 a	0.0020 a

^1^ Mean values (Ave) are subsampling of three replications of one treatment.

^2^Data followed by the same letter is not significantly different between treatments (P ≤0.05, SPSS).

OUT: Operational Taxonomic Units; Ace: community richness; Chao: community richness; Shannon: community diversity; Simpson: community diversity.

### MiSeq-pyrosequencing results and bacterial community structures

**Fig 1:**
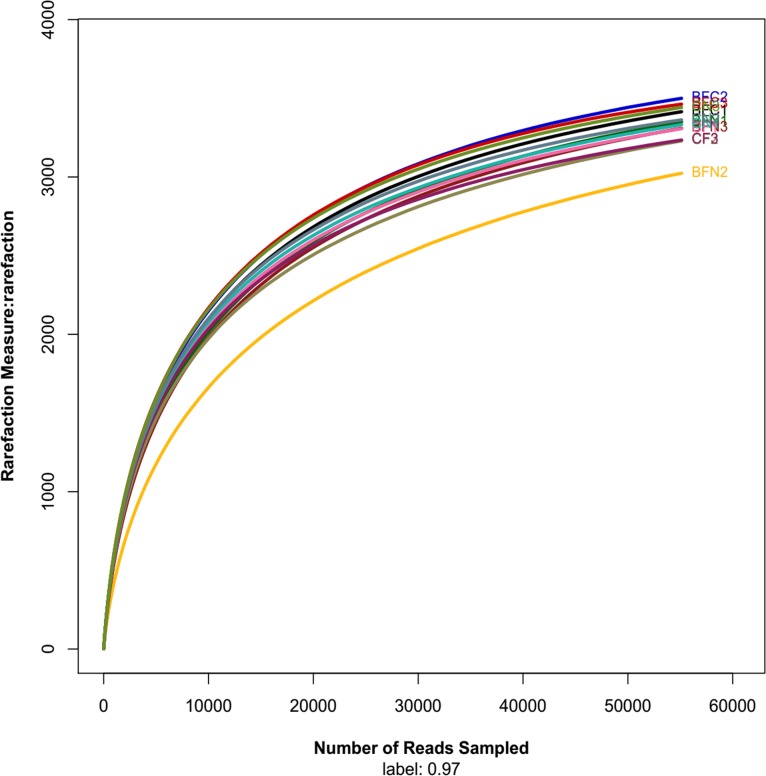
Rarefaction curves based on the 16S rRNA gene sequencing of different treatments.

**Fig 2:**
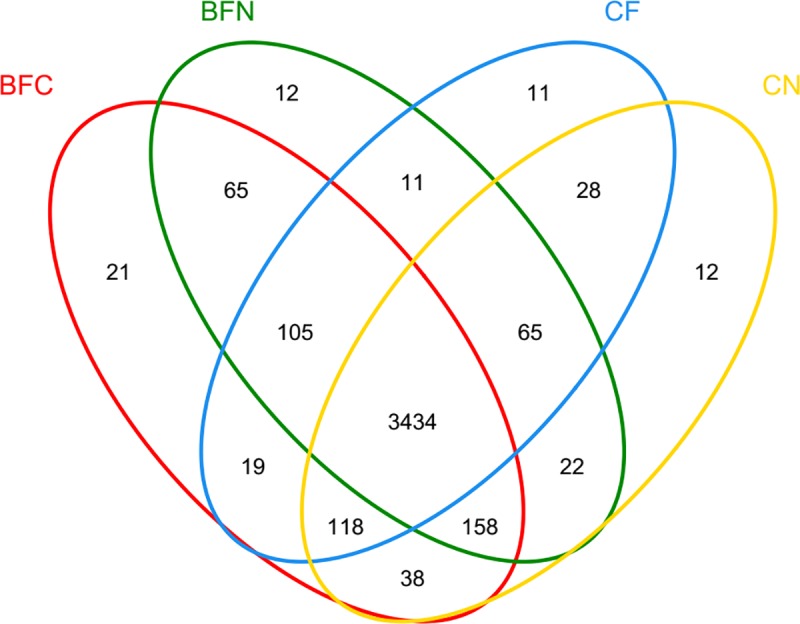
OTU Venn analysis of different treatments.

Rarefaction analysis was used to standardize and compare observed taxon richness between samples and to identify whether the samples were unequally taken [28]. According to the rarefaction curves of different treatments (Fig 1), all of the samples were unequally collected. As shown in the Venn diagram (Fig 2), 3,434 OTUs were common in all test samples among the different treatments. The mustard and oilseed rape treatments had identical subsets (3,762 OTUs), indicating that biological treatments played similar roles in changing microbial populations, while chemical treatments had little effect on bacterial OTUs.

**Fig 3:**
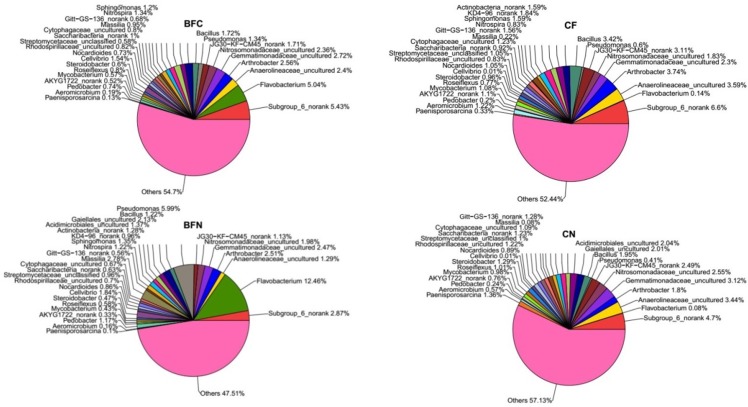
Relative abundance of different bacterial community structures at the genus level in different treatments.

The bacteria from our four soil samples were similar in diversity but different in abundance. An overview of the development in the bacterial community structure during the treatment period is shown at the genus level in Fig 3. In total, 30 identified genera were observed; common taxa were *Arthrobacter* (1.8%-3.74%), *Gemmatimonadaceae*_uncultured (2.3%-3.12%), *Nitrosomonadaceae*_uncultured (1.83%-2.55%), *Bacillus* (1.22%-3.42%), *Gaiellales*_uncultured (2.01%-2.13%), *Acidimicrobiales*_uncultured (1.59%-2.37%), and *Actinobacteria*_norank (1.59%-1.28%). The amounts of *subgroup_6_norank* (2.87%-6.6%), *Flavobacterium* (0.08%-12.46%), *Pseudomonas* (0.41%-5.99%) and *Cellvibrio* (0.01%-1.84%) were different between each treatment. *Flavobacterium* content occupied 12.46% of the total bacteria in the BFN treatment, while it was 5.04%, 0.14% and 0.08% in BFC, CF and CN, respectively. *Subgroup_6_norank* was 6.6% in the CF treatment, but it decreased in the BFC, BFN and CF treatments. The *Pseudomonas* content was 0.41% in the CN treatment, but it increased to 5.99% in the BFN treatment. Each treatment had different strong and weak species.

**Fig 4:**
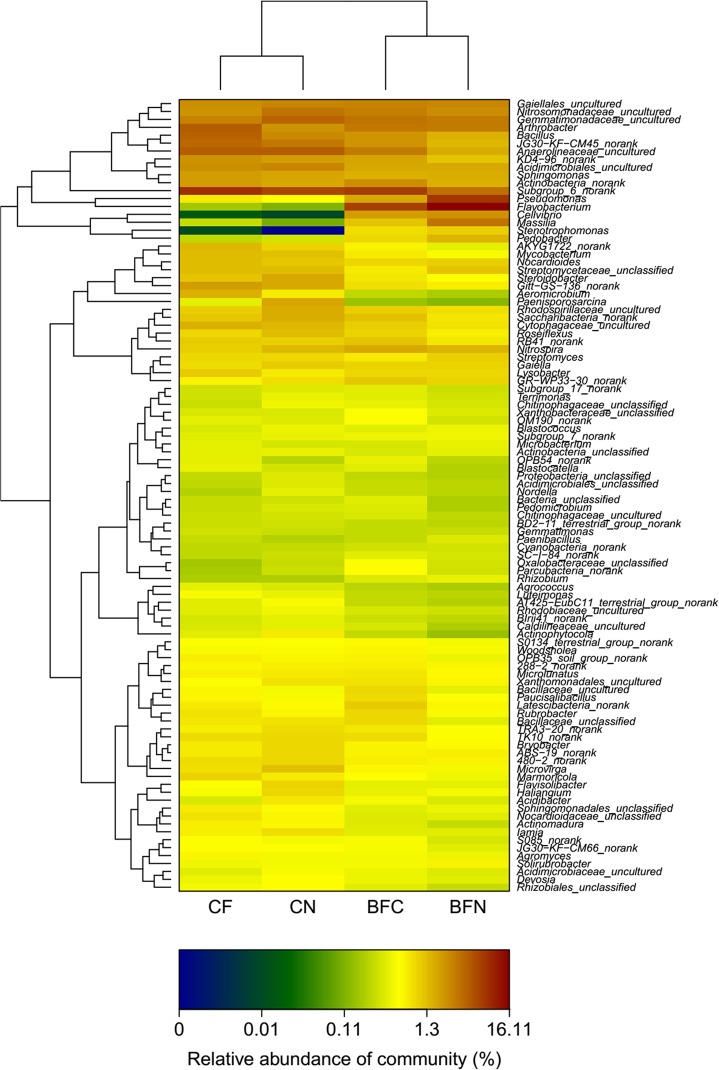
Bacterial community Heatmap analysis of different treatments.

Further Heatmap analysis was used to identify the relative bacterial communities. The bacterial community structures of the four treatments (BFN, BFC, CF and CN) were divided into four clusters according to a similarity comparison (Fig 4). BFN and BFC were clustered into one group, which was different from the group to which CF and CN belonged. This suggested that the BFN and BFC treatments had a similar community structure, and were different from that of the CF and CN community structures. The four clusters were divided into four groups obviously, which suggested clear differences in community structures among the four clusters. The color of the relative abundance of the community varied from blue to red, indicating that it changed from low to high. The abundance of *Stenotrophomonas*, *Massilia* and *Cellvibrio* were clearly higher in BFC and BFN treatments than that in the CF and CN treatments. However, *Subgroup_6_norank* and *Actinophytocola* in the CF and CN treatments were greater abundance than in the BFN treatment.

### Effect of environmental factors on the distribution of bacterial communities

**Fig 5:**
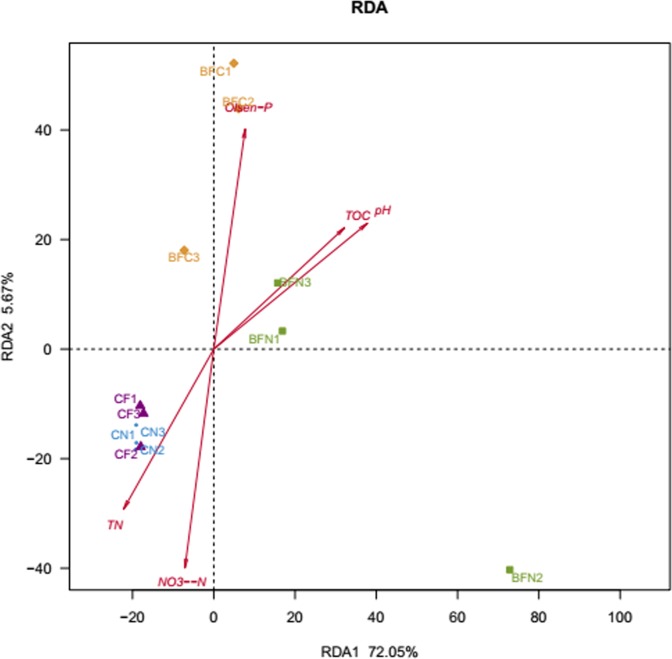
The effect of environmental factors on the distribution of bacterial communities. (TOC: total organic matter; Olsen-p: available P; TN: total N; NO_3_^-^-N: nitrate N)

In total, 77.72% of the species variance was explained by the two axes. The RDA analysis (Fig 5) showed that the bacterial communities in the different treatments were clearly distinctive. There were strong similarities between samples originating from the CF and CN treatments, which are both in the third quadrant. The BFC treatment was similarly close to the positive y-axis, while the BFN treatment was in the first quadrant. These results indicated that the BFC and BFN treatments had similar bacterial communities. Five environmental factors were chosen to compare their effects on the different treatments. They were selected according to the significant differences in the statistical analysis showed by Table 1. The red arrows represent environmental factors. Their positive and negative correlation with the different treatments are represented by angles between treatments and environmental factors (e.g., acute angle: positive correlation; obtuse angle: negative correlation; rectangular: no correlation). Total organic carbon (TOC) and pH clearly correlated with the BFN treatment; soil pH mostly affected the bacterial communities of BFN treatments. The bacterial community of the BFC treatment was greatly affected by available P. This result is consistent with the available P content, which was the highest in the BFC treatment. Compared to the BFC and BFN treatments, soil nitrate N and total nitrogen was more strongly associated with CF and CN treatments.

## Discussion

### Changes in soil physical and chemical properties

In our experiment, soil organic matter and Olsen-P contents of the BFN and BFC treatments were clearly higher than those of the CN and CF treatments, which suggested that the *Brassica* residues acted as a green manure to alter the physicochemical properties of soil that was previously subject to eggplant monocropping [31]. Costa and Crusciol demonstrated that crop rotation can increase the input of organic C in the soil [32], which led to enhance soil fertility. We found similar results in our research that the TOC of BFN treatment was 2.61 and 1.93 times higher than that of CN and CF treatments, respectively (Table 1). Our results showed that NO_3_^-^-N of BFC and BFN was significantly lower than that of the CN and CF treatments, which could be the reason why mustard and oilseed rape treatments weakened nitrification. This result is the same as Tian’s and Kramer’s findings, which indicated that composting decreased the nitrification potential in soil [1, 33]. Some studies showed that soil subject to monocropping in a greenhouse leads the soil to acidification [34]. In our experiment, the soil pH rose slightly in rotational treatments, but there were no significant differences between treatments. These results showed that cover crops could be successfully used for reducing external inputs without creating negative changes in the soil. Therefore, that rotation of eggplant with *Brassica* vegetables is a good way to improve soil physicochemical properties of during continuous planting in a greenhouse.

### Bacterial diversity and composition

In the past, the Sanger and other manual sequencing methods were applied to DNA sequence analysis, but they have been replaced by automatic sequencing. Compared with traditional methods, Illumina MiSeq automatic sequencing incorporates indexed primers to enable the characterization of multiple microbial communities with large quantities of high quality sequenced data and has revealed DNA from organisms that exist at relatively low abundances [35, 36]. In this study, the data from MiSeq-pyrosequencing were more reliable as they improved the knowledge of soil bacteria population diversity and structure. In the MiSeq-pyrosequencing method, the rarefaction curve showed the taxon richness of each sample and identified whether a sample was unequally collected [28]. At a cutoff of 3%, the bacterial community of our rarefaction analyses (Fig 1) showed that the rarefaction curve became relatively flat, implying that the sequencing data amount was reasonable. Ace, Chao, Shannon-Weaver and Simpson indices were used to analyze the effects of the biological and chemical treatments on soil bacterial community diversity (Table 2). Ace and Chao indices indicated that the highest bacterial richness was in biological treatments (BFN and BFC), while the lowest richness was found in the chemical soil fumigated treatment (CF). The Shannon-Weaver and Simpson indices showed that CN treatment community diversity was the lowest, while BFN treatment diversity was the highest (Table 2). The diversity of the CF treatment was similar to the BFC treatment. These results from the high throughput sequencing analysis of MiSeq suggested that *Brassica* vegetable and eggplant rotational treatments (BFC and BFN) increased bacterial richness and diversity in soil that was previously subject to greenhouse monocropping. Soil bacterial diversity can indicate soil ecosystem stability and change of soil fertility. Shiomi found that it was difficult for pathogens to grow and reproduce in the high bacterial diversity soil [37] because of bacterial antagonism and competition [38]. In this experiment soil bacterial diversity of rotational treatment was enhanced, which could change soil microecological and alleviate soilborne disease incidence in continuous eggplant cropping.

In total, 35 genera were identified in these twelve samples (Fig 3). Many of the bacteria genera were similar; however, some differences were observed between the treatments. For example, no *Flavobacteria* existed in the CF and CN treatments; however, it emerged in BFC and BFN and occupied 5.04% and 12.94% of the community, respectively. Some studies have reported that *Flavobacteria* grow stronger in sediments, lakes, frozen, and soils rich with organic matter [39]. The BFC and BFN treatments had more soil organic matter than the control, which could be the reason that *Flavobacteria* existed in these two rotational treatments. Crawford & Mohn (1985) used *Flavobacterium* to remove pentachlorophenol from a variety of contaminated soils, including actual waste-dump soils [40]. Many bacterial genera, including *Actinomycetes* and *Pseudomonas* spp., have been reported to inhibit soil-borne pathogens and promote the growth of the plants [21, 41]. In our experiment, *Pseudomonas* thrived very well in rotational treatments and especially in the BFN treatment. Therefore, BFN treatments could be beneficial for overcoming continuous soil-borne diseases and improved soil physical and chemical properties. Moreover, Zhang et al. reported that a mixture of neem cake and *Pseudomonas fluorescens* could control the *Fusarium* wilt of bananas [42]. *Pseudomonas* was used as an antagonist, which suppressed numerous plant pathogens and promoted plant growth [43]. These results suggested that a *Brassica* vegetable-eggplant rotation could help continuous eggplant systems overcome soil-borne disease. Levels of *Paenisporosarcina* and *Aeromicrobium* were lower in the BFC and BFN treatments as opposed to the CN treatment. *Aeromicrobium* produces antibiotics, which caused damage to the ecosystem when discharged into the environment [44]. Thus, the relative abundance of harmful micro-organisms was reduced in crop rotation.

Additionally, as demonstrated in the bacterial community Heatmap (Fig 4), the abundance of *Stenotrophomonas*, *Massilia*, and *Cellvibrio* was different between the use of biomaterials (BFC and BFN) and chemical materials and the control (CF and CN, respectively), particularly in the case of *Stenotrophomonas*. There was greater abundance of the above-mentioned species in the BFC and BFN treatments compared to the CF and CN treatments. In some niches, *Stenotrophomonas* can degrade a variety of xenobiotic compounds [45], such as chlorpyrifos, methyl parathion, methyl paraoxon, diazinon, phoxim, parathion, profenofos, and triazophos [46]. Thus, *Stenotrophomonas* plays a significant role in the bioremediation of polluted sites, especially those with Cr [47]. They could promote plant growth or be biological control agents of plant pathogens that rely on their phytohormones [48] and chitinolytic [49]. Moreover, *Massilia* sp. WG5 can be applied for bioremediation in PHE-contaminated soil [50]. Additionally, Maya reported that the colonization of *Massilia* on seed coat, radicle, or roots protected against infection by the soil-borne plant pathogen *Pythium aphanidermatum* at the plant developmental stage [51]. *Cellvibrio* are involved in xyloglucan saccharification [52, 53], which can control the development and metabolism of plants and regulate the activity of plant immunomodulatory function. One of the major functional components of Xyloglucan is to prevent plants from becoming sunburned [54]. These reports confirmed that the *Brassica* vegetable-eggplant rotation strongly altered the diversity and composition of bacterial communities in greenhouse soil with eggplant monocropping.

### Relationships between environmental factors and bacterial communities

The RDA diagram (Fig 5) demonstrated a significant distinction in the bacterial communities of the biological and chemical/common treatments. The interaction between soil physical-chemical properties and bacterial community was shown in RDA. Available P, total organic C and pH were the dominant factors that influenced the soil bacterial community structure. This may be because adding vegetable residues to the soil could have regulated nutrient content in the soil, which further affected the soil bacterial community structure [55]. Adding vegetable residue into the soil is similar to adding green manure. High biomass *Brassica napiformis* selected for the BFN treatment was effective in influencing the soil bacterial community. Omirou et al. noted that the effects of broccoli plant residue on microbial communities mirrored the effect of soil enrichment with fresh and decomposed organic matter, rather than a directly releasing toxic isothiocyanates from hydrolyzed glucosinolates [56]. Other research has shown that soil enrichment with organic matter increased soil bacterial diversity [3, 20, 57]. We found similar results: bacterial diversity of rotational treatments (BFN and BFC) was significantly higher than the soil pesticide treatment (CF) and control. Dominant species of the BFN treatment, such as *Flavobacteria*, have been reported to grow more strongly in sediments, frozen lakes and organic-rich soil [39]; meanwhile, the organic matter of the BFN treatment was higher than the other treatments in the experiment (Table 1). Considering that microbial populations rapidly respond to environmental variations [58], the improvement of soil physical-chemical properties in the BFC and BFN treatments led to the change in soil diversity and structure. Soil microorganisms are critical to the soil ecosystem because of their significant role in regulating soil nutrients and carbon cycling via fundamental ecological processes, such as mineralization and decomposition [59].

In addition, the experiment was located in a high latitude province. Crops are cultivated once a year in this region. Eggplant is typically cultivated from the beginning of May (or June, in the case of land cultivation) to the end of August. After eggplant harvesting in this area, growth time and temperature in greenhouses are suitable for planting *Brassica* vegetables. Mustard and oilseed rape were planted in this experiment, which contain high glucosinolates and grow 45–50 days at suitable temperatures of 20–25°C. Roots of mustard are usually used for pickled vegetables in this area, and their leaves can be incorporated into soil. Therefore, mustard rotation with eggplant would be and the most economically sustainable practice for the farmers in this province. This, to some extent, could help farmers overcome the challenges of eggplant monocropping in greenhouses.

## Conclusion

This study investigated the effects of a *Brassica* vegetable-eggplant rotation on the physiochemical properties of soil and bacterial diversity and community structures. The physical and chemical properties of soil under eggplant monocropping in greenhouses were improved by mustard and oilseed rape rotation. Soil treated with mustard or oil seed improved soil bacterial diversity, and as a result, the soil’s bacterial community structure was altered. At the same time, the proportion of beneficial bacterial species increased within rotational treatments compared with the chemical fumigated treatment and control. These results illustrate that rotational methods would provide a better way of solving the problems of eggplant monocropping.

## Supporting information

S1 Table Soil physicochemical characteristics of different treatments. Mean values (n = 3) for each treatment that are followed by the same letter are not significantly different (P ≤0.05) between treatments in the same column.
(DOCX)

S2 Table Bacterial diversity indices of different rotational treatments. ^1^ Mean values (Ave) are subsampling of three replications of one treatment. ^2^Data followed by the same letter is not significantly different between treatments (P ≤0.05, SPSS). OUT: Operational Taxonomic Units; Ace: community richness; Chao: community richness; Shannon: community diversity; Simpson: community diversity.
(DOCX)
