# Supplementary material for: Changes in soil bacterial community structure as a result of incorporation of Brassica plants compared with continuous planting eggplant and chemical disinfection in greenhouses
Source: PLoS One. 2017 Mar 27;12(3):e0173923. doi: 10.1371/journal.pone.0173923 (PMC5367839; doi:10.1371/journal.pone.0173923)
Supplement: S1 Table — Mean values (n = 3) for each treatment that are followed by the same letter are not significantly different (P ≤0.05) between treatments in the same column. (DOCX) [file pone.0173923.s001.docx]

**Table 1** **Soil physicochemical characteristics of different treatments.**

| Treatments | pH | Organic matter | Organic C | Total N | Total P | Available P | Available K | Ammonium N | Nitrate N |
| --- | --- | --- | --- | --- | --- | --- | --- | --- | --- |
|  |  | （g•kg^-1^) | (g•kg^-1^) | (g•kg^-1^) | ( g•kg^-1^) | (mg•kg^-1^) | (mg•kg^-1^) | (mg•kg^-1^) | (mg•kg^-1^) |
| CN | 7.07 b | 20.42 b | 11.84 b | 1.41 ab | 2.33 b | 128.63 c | 187.69 a | 14.08 a | 32.77 a |
| BFN | 7.47 a | 53.30 a | 30.92 a | 1.31 b | 2.63 a | 138.53 b | 186.83 a | 13.68 a | 28.91 b |
| BFC | 7.39 a | 43.67 a | 25.33 a | 1.31 b | 2.17 c | 182.67 a | 179.19 b | 13.92 a | 14.03 c |
| CF | 7.07 b | 27.62 b | 16.02 b | 1.44 a | 2.30 b | 131.57 c | 187.18 a | 14.02 a | 32.83 a |

Note: Mean values (n=3) for each treatment that are followed by the same letter are not significantly different (P ≤0.05) between treatments in the same column.
