# Supplementary material for: Changes in soil bacterial community structure as a result of incorporation of Brassica plants compared with continuous planting eggplant and chemical disinfection in greenhouses
Source: PLoS One. 2017 Mar 27;12(3):e0173923. doi: 10.1371/journal.pone.0173923 (PMC5367839; doi:10.1371/journal.pone.0173923)
Supplement: S2 Table — 1 Mean values (Ave) are subsampling of three replications of one treatment. 2Data followed by the same letter is not significantly different between treatments (P ≤0.05, SPSS). OUT: Operational Taxonomic Units; Ace: community richness; Chao: community richness; Shannon: community diversity; Simpson: community diversity. (DOCX) [file pone.0173923.s002.docx]

**Table 2** **Bacterial diversity indices of different rotational treatments.**

| Treatments | Reads | 0.97 | | | | |
| --- | --- | --- | --- | --- | --- | --- |
|  |  | OTU | Ace | Chao | Shannon | Simpson |
| BFC1 | 55129 | 3415 | 3773 | 3801 | 6.95 | 0.0029 |
| BFC2 |  | 3500 | 3854 | 3860 | 7.00 | 0.0029 |
| BFC3 |  | 3463 | 3753 | 3740 | 7.09 | 0.0021 |
| BFC_Ave_^1^ |  | 10378 | 3793 a^2^ | 3800 a | 7.01 a | 0.0026 a  0.0039 |
| BFN1 |  | 3350 | 3773 | 3744 | 6.78 |  |
| BFN2 |  | 3024 | 3629 | 3607 | 5.87 | 0.015 |
| BFN3 |  | 3310 | 3738 | 3758 | 6.76 | 0.0036 |
| BFN_Ave_ |  | 9684 | 3713b | 3703 a | 6.47 b | 0.0076 a |
| CF1 |  | 3307 | 3684 | 3724 | 6.98 | 0.0024 |
| CF2 |  | 3230 | 3682 | 3767 | 6.86 | 0.0033 |
| CF3 |  | 3235 | 3581 | 3634 | 6.96 | 0.0027 |
| CF_Ave_ |  | 9772 | 3649 b | 3708 a | 6.93 ab | 0.0028 a |
| CN1 |  | 3333 | 3710 | 3751 | 7.04 | 0.0020 |
| CN2 |  | 3363 | 3699 | 3720 | 7.03 | 0.0022 |
| CN3 |  | 3442 | 3771 | 3814 | 7.09 | 0.0019 |
| CN_Ave_ |  | 10138 | 3727 ab | 3762 a | 7.05 a | 0.0020 a |

^1^ Mean values（Ave）are subsampling of three replications of one treatment. ^2^Data followed by the same letter is not significantly different between treatments (P ≤0.05, SPSS). OUT: Operational Taxonomic Units; Ace: community richness; Chao: community richness; Shannon: community diversity; Simpson: community diversity.
